# Microclimatic Influences on Soil Nitrogen Dynamics and Plant Diversity Across Rocky Desertification Gradients in Southwest China

**DOI:** 10.3390/plants14081251

**Published:** 2025-04-20

**Authors:** Qian Wu, Chengjiao Rao, Wende Yan, Yuanying Peng, Enwen Wang, Xiaoyong Chen

**Affiliations:** 1 College of Resources and Environmental Engineering, Anshun University, Anshun 561000, China; 2 College of Life Science and Technology, Central South University of Forestry and Technology, Changsha 410004, China; raorao199709@163.com (C.R.); t20001421@csuft.edu.cn (W.Y.); 3 National Engineering Laboratory for Applied Forest Ecological Technology in Southern China, Changsha 410004, China; 4 College of Arts and Sciences, Lewis University, Romeoville, IL 60446, USA; pengyu@lewisu.edu; 5 College of Arts and Sciences, Governors State University, University Park, IL 60484, USA

**Keywords:** soil active nitrogen, microclimate types, karst ecosystem, rocky desertification, biodiversity

## Abstract

Soil active nitrogen (N) fractions are essential for plant growth and nutrient cycling in terrestrial ecosystems. While previous studies have primarily focused on the impact of vegetation restoration on soil active nitrogen in karst ecosystems, the role of microclimate variation in rocky desertification areas has not been well explored. This study investigates soil active nitrogen fractions and key biotic and abiotic factors across four grades of rocky desertification—non-rocky desertification (NRD), light rocky desertification (LRD), moderate rocky desertification (MRD), and intense rocky desertification (IRD)—within two distinct microclimates: a dry-hot valley and a humid monsoon zone in the karst region of Guizhou Province, China. We evaluate soil organic carbon (SOC), total nitrogen (TN), total phosphorus (TP), soil nitrate nitrogen (NO_3_^−^-N), ammonium nitrogen (NH_4_^+^-N), microbial biomass nitrogen (MBN), soluble organic nitrogen (SON), and plant diversity. Results showed that SOC, TN, and TP were significantly higher in IRD areas. Soil NO_3_^−^-N, MBN, and SON initially decreased before increasing, with consistent MBN growth in the dry-hot valley. NH_4_^+^-N did not differ significantly under NRD but was higher in the dry-hot valley under LRD, MRD, and IRD. The dry-hot valley had higher MBN and SON across most desertification grades. Microclimate significantly influenced soil active N, with higher levels in the dry-hot valley under LRD and MRD conditions. Plant diversity and regeneration varied markedly between the microclimates. In the dry-hot valley, *Artemisia* dominated herbaceous regeneration, especially in MRD areas. Conversely, the humid monsoon zone showed more diverse regeneration, with *Artemisia* and *Bidens* prevalent in MRD and NRD grades. Despite declining plant diversity with desertification, the humid monsoon zone displayed greater resilience. These findings highlight the role of microclimate in influencing soil nitrogen dynamics and plant regeneration across rocky desertification gradients, offering insights for restoration strategies in karst ecosystems.

## 1. Introduction

Climate change and desertification are among the most pressing environmental challenges of our time. These interconnected phenomena threaten ecosystems globally, with severe implications for biodiversity, soil health, and water resources, impacting the livelihoods of millions [1,2]. The increasing frequency of extreme weather events, shifts in temperature, and erratic precipitation patterns are transforming landscapes, particularly in fragile environments like karst regions, which are highly sensitive to environmental stressors [3,4].

A critical manifestation of environmental degradation in karst landscapes is “rocky desertification”. This process, driven by deforestation, unsustainable agriculture, and overgrazing, results in vegetation loss, soil fertility decline, and exposure of bedrock. Often described as “the cancer of the Earth”, rocky desertification represents a rapid and irreversible decline in ecosystem functionality, with profound consequences for both local and global environmental health [5,6].

Karst landscapes, characterized by features such as sinkholes, caves, and exposed rock surfaces, occupy approximately 22 million square kilometers—around 12% of the Earth’s land area and 20% of its ice-free land [7,8]. These terrains, formed by the dissolution of soluble bedrock like limestone and dolomite, are ecologically significant yet highly vulnerable. In Southwest China’s Guizhou Province, one of the world’s most extensive karst regions, rocky desertification poses a severe challenge due to shallow soils, soil erosion, and nutrient depletion, exacerbating local poverty and hindering natural restoration efforts [5,9,10]. Despite restoration initiatives since the 1980s achieving some success, challenges remain, particularly regarding how microclimatic variability affects soil active nitrogen fractions and plant diversity in these fragile ecosystems [9,11]. Plant diversity in karst ecosystems is shaped by a complex interplay of microclimatic variables, soil properties, and human activities. These landscapes are biodiversity hotspots, harboring unique and endemic species due to their heterogeneous topography and soil conditions [12,13]. However, gradients of rocky desertification alter soil moisture, nutrient availability, and microhabitat conditions, often leading to declines in species richness and diversity [13,14]. Severe rocky desertification further exacerbates these effects, reducing water retention and depleting nutrients, thereby intensifying ecological degradation [15].

Nitrogen, a crucial nutrient for plant growth, plays a pivotal role in ecosystem productivity and health. Soil nitrogen exists in various forms, including ammonium (NH_4_^+^-N), nitrate (NO_3_^−^-N), microbial biomass nitrogen (MBN), and soluble organic nitrogen (SON). These active nitrogen fractions, although constituting only a small proportion of total soil nitrogen, are highly mobile and bioavailable, directly influencing nutrient cycling and plant growth [16,17,18]. SON is vital for nutrient dynamics, serving both as an essential component of the nitrogen cycle and a significant pathway for nitrogen loss [19,20]. Similarly, MBN, derived from soil microorganisms, plays a central role in nitrogen transformations, linking organic matter decomposition to plant nutrient availability [21].

Microclimatic factors such as temperature, humidity, and radiation significantly influence soil nitrogen dynamics and plant community structure in karst ecosystems. Variations in these factors along rocky desertification gradients can intensify environmental stressors or create microrefugia that support higher biodiversity [22]. Understanding how microclimatic variability affects soil nitrogen dynamics and plant diversity is essential for developing targeted restoration strategies to enhance ecosystem resilience.

Although previous studies on rocky desertification in Southwest China have explored soil nutrient changes under various restoration and land-use practices, most have focused on single climatic regions. For instance, Cheng et al. [9] reported that soil nitrogen (N) storage in afforested lands was significantly lower than in adjacent croplands in a typical karst area under a humid monsoon climate. Additionally, soil total N concentrations were significantly higher in shrublands and forests compared to croplands and grasslands, while the gross rates of nitrogen mineralization and nitrification were significantly lower in grasslands than in croplands [20]. There is a notable lack of comparative research examining the influence of different microclimates on soil properties and active nitrogen fractions across varying rocky desertification gradients [23].

To address this knowledge gap, our study investigates two contrasting climate types in Anshun City, Guizhou Province of China: the dry-hot valley and humid monsoon climates. We hypothesize that (1) soil environmental factors, active nitrogen fractions, and plant diversity differ significantly across rocky desertification gradients, and (2) the dry-hot valley climate exerts a stronger influence on these parameters than the humid monsoon climate. The objectives of this study were to: (1) examine the distribution patterns of soil environmental factors and plant diversity across varying rocky desertification gradients, and (2) identify the key factors driving variations in active nitrogen fractions and plant diversity within these distinct climate types.

## 2. Results

### 2.1. Soil Properties and Enzyme Characteristics Across Four Desertification Grades and Three Soil Layers in Two Distinct Regions

This study revealed significant variations in soil physicochemical properties and enzyme activities across different grades of rocky desertification in the dry-hot valley and humid monsoon climates (Table 1). Soil moisture content (SMC), pH, soil organic carbon (SOC), total nitrogen (TN), total phosphorus (TP), urease activity, and protease activity were examined across four desertification grades (NRD, LRD, MRD, and IRD) and three soil layers.

In the dry-hot valley, SMC generally increased with soil depth. Significant differences were observed between NRD and MRD or IRD in the A layer, while similar patterns were noted between NRD, LRD, and higher grades in the B and C layers (*p* < 0.05). However, no significant differences in SMC were detected between the A and B layers for MRD (*p* > 0.05). Soil pH values decreased with increasing desertification severity and depth, following the trend NRD > LRD > MRD > IRD. Concentrations of SOC, TN, and TP exhibited variable patterns, generally increasing in the IRD grade. SOC peaked at 30.83 g/kg, TN at 3.32 g/kg, and TP at 1.92 g/kg in the IRD grade. Enzyme activities for urease and protease were also highest in the IRD grade, reflecting enhanced nutrient cycling in more severe desertification conditions (Table 1).

In the humid monsoon climate, SMC values were significantly higher than those in the dry-hot valley and increased with desertification severity. Notable differences were found between IRD and NRD or LRD in all three soil layers (*p* < 0.05). The highest SMC was observed in the IRD grade (27.08%) compared to 26.18% in the dry-hot valley. Soil pH followed a similar trend to that in the dry-hot valley, with the lowest values in the MRD and IRD grades, ranging from 7.37 to 7.52. SOC was highest in NRD (42.06 g/kg) but lowest in LRD (20.36 g/kg). Similarly, TN and TP levels were elevated in the IRD grade, with TN reaching 3.47 g/kg and TP peaking at 1.92 g/kg. Enzyme activities were higher in the humid monsoon region, with NRD recording the highest urease activity at 1.18 mg/g (Table 1).

### 2.2. Soil C/N, C/P, and N/P Ratios Across Four Grades of Rocky Desertification and Three Soil Layers in Two Regions

In the humid monsoon climate zone, the C/N, C/P, and N/P ratios generally decrease as the severity of rocky desertification increases and with greater soil depths. For instance, in non-rocky desertified areas (NRD), the C/N ratio decreases from 7.42 ± 0.38 at the shallowest soil depth (A) to 3.85 ± 0.38 at the deepest layer (C). This trend indicates a reduction in soil organic carbon (SOC) relative to nitrogen (TN) with increasing depth. Similarly, the C/P ratio declines from 40.69 ± 9.10 at depth A to 15.40 ± 3.85 at depth C, suggesting a diminishing organic carbon availability relative to phosphorus (TP) as soil depth increases. The N/P ratio follows a similar pattern, with a decrease observed across depths, pointing to altered nutrient dynamics under more severe desertification conditions. In contrast, the dry-hot valley climate zone exhibits relatively stable C/N and N/P ratios across the different rocky desertification grades and soil layers. Specifically, in non-rocky desertified areas (NRD), the C/N ratios remain consistent across the three depths (e.g., 12.10 ± 0.59 at depth A and 12.81 ± 1.18 at depth C). However, the C/P ratio in this zone increases as rocky desertification severity increases, with values rising from 42.40 ± 15.06 at depth A to 48.78 ± 21.81 at depth C.

The humid monsoon region demonstrates more pronounced effects of rocky desertification on nutrient ratios, particularly with decreasing C/N and C/P ratios, whereas the dry-hot valley region maintains more stable nutrient dynamics, with an increase in the C/P ratio as desertification severity intensifies. The observed differences highlight the varying responses of soil nutrient cycling to climatic and land degradation factors in different regions (Table 2).

### 2.3. Variations in Herb and Shrub Regeneration Across Rocky Desertification Grades and Microclimates in Two Climate Zones

Plant diversity and regeneration patterns showed distinct differences between the two microclimates—the dry-hot valley (Zone 1) and the humid monsoon region (Zone 2)—across the four grades of rocky desertification (Table 3). In Zone 1, herbaceous regeneration was predominantly dominated by *Artemisia*, particularly in the moderate rocky desertification (MRD) grade, where it accounted for 52% of the total herbaceous regeneration, suggesting reduced species diversity in response to increasing desertification. Shrub regeneration in this zone also exhibited a strong *Artemisia* presence, with notable contributions observed in the light rocky desertification (LRD) grade. Conversely, Zone 2 exhibited more extensive plant regeneration, with a more even distribution of herbaceous and shrub species across the desertification gradients. In this region, *Artemisia* and *Bidens* were notably abundant in the MRD and non-rocky desertified (NRD) grades. Although plant diversity and regeneration declined with increasing desertification severity in both regions, Zone 2 demonstrated greater resilience, maintaining higher species richness and more stable regeneration patterns across the gradients, suggesting stronger adaptive capacity under degrading conditions. Additional noteworthy genera included *Vicia* and *Clematis*, with *Vicia* being particularly prominent in Zone 1 under intensive rocky desertification (IRD) conditions. These findings highlight the contrasting vegetation responses between climate zones and emphasize the importance of microclimatic conditions in shaping plant regeneration dynamics.

### 2.4. Effects of Rocky Desertification and Soil Depth on Soil Active Nitrogen in Two Climate Regions

**Figure 1:**
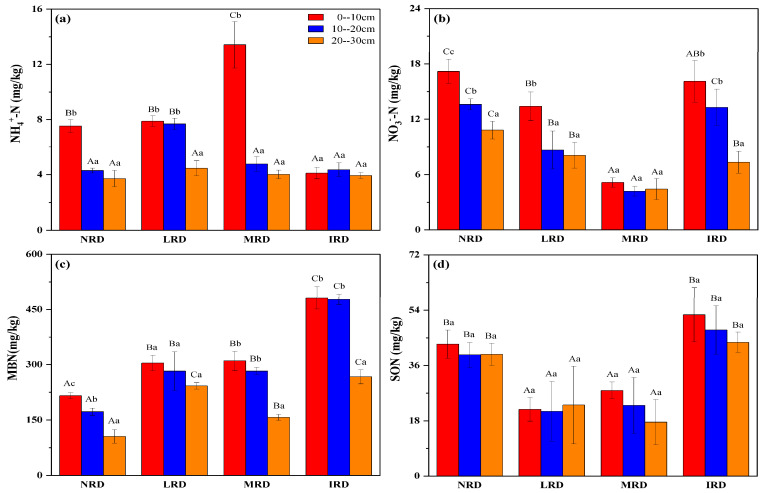
Characteristics of soil active nitrogen in the dry-hot valley climate zone, with NH_4_^+^-N concentration (**a**), NO_3_-N concentration (**b**), MBN concentration (**c**) and SON concentration (**d**). Capital letters (A, B, C) indicate significant differences among the four grades of rocky desertification, while lowercase letters (a, b, c) indicate significant differences within the soil layers (A, B, and C). The figure shows soil NH_4_^+^-N and NH_3_-N concentrations, microbial biomass nitrogen (MBN), and soil organic nitrogen (SON) across three soil layers in four grades of rocky desertification; non-rocky desertification (NRD), light rocky desertification (LRD), moderate rocky desertification (MRD), and intense rocky desertification (IRD).

**Figure 2:**
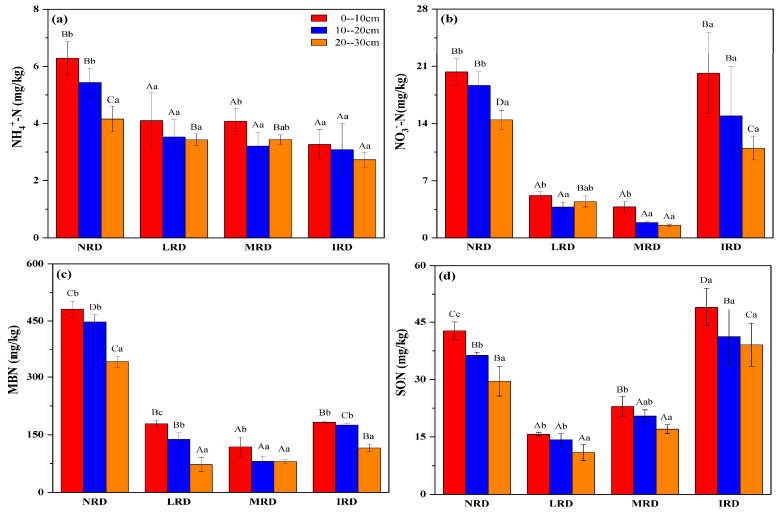
Characteristics of soil active nitrogen in the humid monsoon climate zone, with NH_4_^+^-N concentration (**a**), NO_3_-N concentration (**b**), MBN concentration (**c**) and SON concentration (**d**). Capital letters (A, B, C) indicate significant differences among the four grades of rocky desertification, while lowercase letters (a, b, c) indicate significant differences within the soil layers (A, B, and C). Figure shows soil ammonium nitrogen (NH_4_^+^-N), nitrate nitrogen (NH_3_-N) concentrations, soil microbial biomass nitrogen (MBN), and soil organic nitrogen (SON) across three soil layers in humid monsoon climate zones among the four grades of desertification; non-rocky desertification (NRD), light rocky desertification (LRD), moderate rocky desertification (MRD), and intense rocky desertification (IRD). Capital letters (A, B, C) indicate significant differences among the four levels of rocky desertification, while lower-case letters (a, b, c) indicate significant differences within the soil layers (A, B, and C).

The data revealed significant differences in soil active nitrogen dynamics between the dry-hot valley and humid monsoon climate zones (Figure 1 and Figure 2). In the dry-hot valley, N components were highest in the IRD grade, whereas in the humid monsoon zone, the NRD grade consistently showed higher concentrations. These results indicated that the influence of rocky desertification on soil active nitrogen is climate dependent, with distinct patterns observed in arid versus humid regions. Figure 1 illustrated the distribution of soil active nitrogen components across different grades of rocky desertification (NRD, LRD, MRD, and IRD) and soil depths (0–10 cm, 10–20 cm, and 20–30 cm) in the dry-hot valley climate zone. Soil NH_4_^+^-N concentrations showed significant variation among rocky desertification grades (*p* < 0.05). NH_4_^+^-N levels were highest in the surface layer (0–10 cm) for all grades, with IRD exhibiting the greatest concentration (12 mg/kg) compared to lower concentrations in MRD, LRD, and NRD. Within each soil layer, NH_4_^+^-N levels decreased with increasing depth, with IRD consistently displaying the highest concentrations across all depths (Figure 1a). Soil NO_3_^−^-N concentrations followed a similar trend, with significant differences observed among desertification grades. IRD demonstrated the highest NO_3_^−^-N levels (18 mg/kg) in the 0–10 cm layer, significantly surpassing the concentrations in the MRD, LRD, and NRD categories. NO_3_^−^-N concentrations also declined with depth, and IRD maintained the highest levels across all layers (Figure 1b). Microbial biomass nitrogen (MBN) was markedly higher in IRD than in other desertification grades, particularly in the 0–10 cm layer (450 mg/kg). MBN concentrations were significantly lower in MRD, LRD, and NRD, with values decreasing progressively with depth (Figure 1c). Soil organic nitrogen (SON) also exhibited significant differences across rocky desertification grades, with the highest concentrations observed in IRD (54 mg/kg in the surface layer). SON levels were consistently lower in deeper soil layers across all desertification grades (Figure 1d).

Ammonium nitrogen (NH_4_^+^-N) concentrations (Figure 1a) were highest in moderate rocky desertification (MRD) areas at the 0–10 cm depth, followed by intense (IRD), light (LRD), and non-rocky desertification (NRD) areas, with concentrations decreasing substantially with depth across all grades. Similarly, nitrate nitrogen (NO_3_⁻-N) concentrations (Figure 1b) peaked in IRD areas at 0–10 cm and decreased consistently with depth, though IRD maintained relatively higher levels than other grades at all depths. Microbial biomass nitrogen (MBN) (Figure 1c) showed a similar pattern, with the highest concentrations in IRD at the surface layer, declining significantly with depth and across grades. Soil organic nitrogen (SON) (Figure 1d) also followed this trend, with IRD recording the highest concentrations at 0–10 cm, decreasing with depth across all grades. These findings underscore the critical role of rocky desertification severity and soil depth in shaping soil nitrogen dynamics, with active nitrogen fractions being most abundant in surface soils and more severely degraded areas. Figure 2 presented the distribution of soil active nitrogen components in the humid monsoon climate zone. As with the dry-hot valley, significant variations in soil NH_4_^+^-N, NO_3_^−^-N, MBN, and SON were observed among different desertification grades and soil layers. Soil NH_4_^+^-N concentrations were highest in the 0–10 cm layer for all desertification grades, with NRD exhibiting the maximum value (8 mg/kg). NH_4_^+^-N concentrations decreased with depth and were significantly lower in IRD compared to NRD and LRD (Figure 2a). Similarly, NO_3_^−^-N concentrations showed significant differences among grades, with the highest levels in NRD (21 mg/kg) in the surface layer. NO_3_^−^-N concentrations declined sharply with depth in all desertification grades (Figure 2b). MBN exhibited a distinct pattern, with NRD displaying the highest levels (500 mg/kg) in the surface layer. MBN concentrations were significantly lower in LRD, MRD, and IRD, with consistent decreases observed across soil depths. For SON, NRD had the highest concentrations in the 0–10 cm layer (45 mg/kg), followed by LRD, MRD, and IRD (Figure 2c). SON levels were significantly lower in the 10–20 cm and 20–30 cm layers for all desertification grades (Figure 2d).

### 2.5. The Relationships Between Soil Active Nitrogen Fractions and Physicochemical Properties

**Figure 3:**
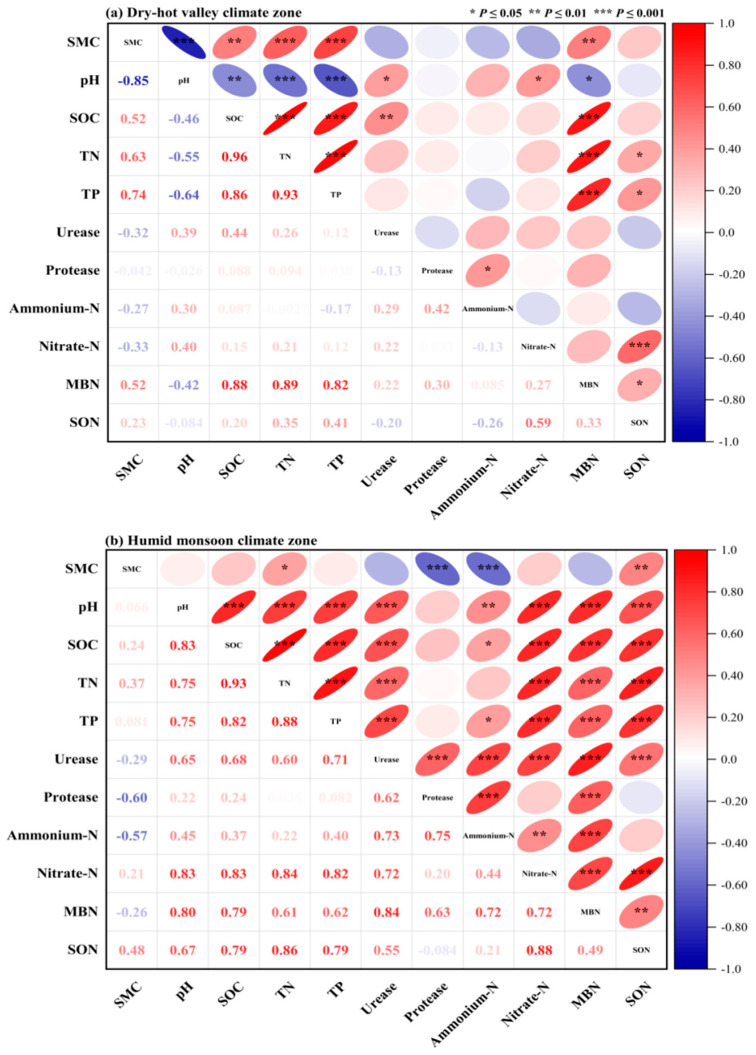
Relationships between soil physicochemical properties and active nitrogen fractions in the two regions. The dry-hot valley climate zone (**a**) and the humid monsoon climate zone (**b**). Statistical significance is indicated as follows: *** *p* < 0.001, ** *p* < 0.01, and * *p* < 0.05.

The relationships between soil active nitrogen fractions and physicochemical properties are shown in Figure 3. In the dry-hot valley climate zone, soil pH exhibited a positive correlation with NH_3_-N (*p* < 0.05) and a negative correlation with microbial biomass nitrogen (MBN) (*p* < 0.05). Additionally, soil organic carbon (SOC) had an extremely significant positive correlation with MBN, while total nitrogen (TN) showed extremely significant positive correlations with both MBN and soluble organic nitrogen (SON). Total phosphorus (TP) also had extremely significant positive correlations with MBN and a positive correlation with SON. Protease activity was positively correlated with NH_4_^+^-N. Furthermore, NH₃-N showed an extremely significant positive correlation with SON, and MBN was positively correlated with SON (Figure 3a).

### 2.6. Comparative Analysis of Soil Active Nitrogen Fractions in the Two Climate Areas

**Figure 4:**
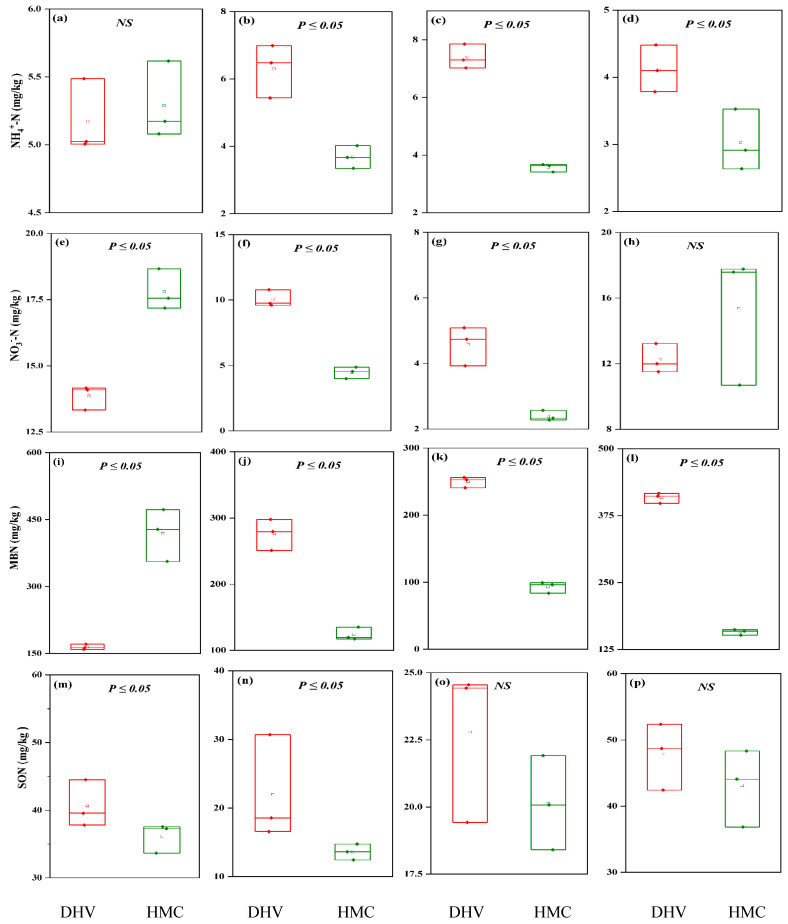
The comparative analysis of soil active nitrogen fractions across different rocky desertification grades in the humid monsoon climate and the dry-hot valley climate region, with NH_4_^+^-N concentration in NRD (**a**), LRD (**b**), MRD (**c**) and IRD (**d**), NO_3_-N concentration in NRD (**e**), LRD (**f**), MRD (**g**) and IRD (**h**), MBN concentration in NRD (**i**), LRD (**j**), MRD (**k**) and IRD (**l**) and SON concentration in NRD (**m**), LRD (**n**), MRD (**o**) and IRD (**p**). Values with *p* ≤ 0.05 indicate significant difference, while NS indicates no significant difference (Wilcoxon signed-rank). DHV and HMC represent the dry-hot valley climate region and the humid monsoon climate region, respectively.

Differences in soil active nitrogen fractions were observed across various grades of rocky desertification in different climatic areas, as determined by the Wilcoxon signed-rank test. In non-rocky desertification (NRD) areas, the soil NH_4_^+^-N levels in dry-hot valley climates were significantly higher than those in humid monsoon climates (*p* < 0.05, Figure 4). In NRD regions, soil NH_3_-N was significantly higher in humid monsoon climates than in dry-hot valley climates. Conversely, in low rocky desertification (LRD) and moderate rocky desertification (MRD) areas, soil NH_3_-N levels were significantly higher in the dry-hot valley climate than in the humid monsoon climate. However, no significant difference was noted in the intermediate rocky desertification (IRD) areas (Figure 4).

In NRD areas, microbial biomass nitrogen (MBN) was significantly higher in the humid monsoon climate than in the dry-hot valley climate. Conversely, in LRD, MRD, and IRD areas, soil MBN levels were significantly higher in the dry-hot valley climate compared to the humid monsoon climate (Figure 4). Furthermore, soil soluble organic nitrogen (SON) was significantly greater in the dry-hot valley climate than in the humid monsoon climate in NRD and LRD areas. However, no significant differences were observed between the climates in MRD and IRD areas (Figure 4). Across the desertification gradients from NRD to IRD, SON levels in the dry-hot valley climate consistently surpassed those in the humid monsoon climate, with significant differences (*p* < 0.05) observed in NRD and LRD areas, but no significant differences in MRD and IRD areas (*p* > 0.05).

## 3. Discussion

### 3.1. Variations in Soil Properties and Enzyme Activities Across Four Desertification Gradients and Three Soil Horizons in Two Contrasting Regions

The findings of this study demonstrate significant variations in soil physicochemical properties and enzyme activities across different desertification grades in the dry-hot valley and humid monsoon climates. These differences align with previous research indicating that climatic conditions and desertification intensity strongly influence soil characteristics [24,25]. In the dry-hot valley, soil moisture content (SMC) generally increased with soil depth, consistent with findings from similar arid environments where reduced evaporation rates at deeper layers result in higher moisture retention [26]. Significant differences in SMC were observed between NRD and MRD or IRD in the A layer, while patterns in the B and C layers indicated a convergence of SMC levels across desertification grades. The absence of significant differences between the A and B layers for MRD suggests that moderate desertification may disrupt typical moisture distribution patterns [27].

Soil pH values exhibited a declining trend with increasing desertification severity and depth, following the pattern NRD > LRD > MRD > IRD. This decline aligns with studies suggesting that intensified desertification enhances soil acidification due to increased leaching of basic cations. Variations in SOC, TN, and TP were similarly influenced by desertification severity, with peak values recorded in the IRD grade. The observed SOC peak at 30.83 g/kg, TN at 3.32 g/kg, and TP at 1.92 g/kg in the IRD grade suggest that nutrient accumulation may result from the deposition of organic material in severely degraded soils [28]. Elevated enzyme activities for urease and protease in the IRD grade further indicate enhanced microbial activity and nutrient cycling in response to desertification stress [29]. In the humid monsoon climate, significantly higher SMC values compared to the dry-hot valley reflect the region’s greater precipitation levels and improved water retention capacity. Increasing SMC with desertification severity is consistent with findings in subtropical regions where vegetation degradation alters soil structure and enhances moisture accumulation in exposed surfaces [30]. The highest SMC in the IRD grade (27.08%) compared to 26.18% in the dry-hot valley further highlights the influence of regional climate factors on moisture dynamics. Soil pH trends in the humid monsoon climate paralleled those in the dry-hot valley, with the lowest values in MRD and IRD grades. SOC levels differed, with the highest concentration recorded in NRD (42.06 g/kg) and the lowest in LRD (20.36 g/kg). The higher SOC content in NRD suggests that less disturbed ecosystems in humid regions accumulate more organic carbon, whereas degraded areas experience substantial carbon loss [27]. Elevated TN and TP levels in the IRD grade align with patterns seen in the dry-hot valley, supporting the notion that severe desertification redistributes nutrient reserves in degraded soils. The highest urease activity in NRD (1.18 mg/g) further highlights the positive association between stable ecosystems and enhanced enzyme function [25].

### 3.2. Variations in Soil Stoichiometric Ratios Across Rocky Desertification Gradients and Soil Layers in Two Regions

The variations in soil carbon-to-nitrogen (C/N), carbon-to-phosphorus (C/P), and nitrogen-to-phosphorus (N/P) ratios across different rocky desertification grades and soil depths reveal distinct nutrient dynamics influenced by climatic conditions. In the humid monsoon region, these ratios generally declined with increasing desertification severity and soil depth, indicating progressive nutrient depletion. This pattern reflects a reduction in soil organic carbon (SOC) relative to nitrogen (TN) and phosphorus (TP), potentially driven by accelerated organic matter decomposition and leaching under humid conditions [31]. While lower C/N and C/P ratios may indicate higher nitrogen and phosphorus availability, in rocky desertification zones, they can also signify a loss of stable carbon pools and a shift toward more labile nutrient forms [9,13]. Such declines in C/N and C/P ratios are characteristic of degraded soils with reduced organic matter quality and nutrient availability [32].

In contrast, the dry-hot valley region displayed relatively stable C/N and N/P ratios across both desertification grades and soil depths, suggesting slower organic matter decomposition and nutrient turnover in arid conditions [28]. The stable C/N ratio likely reflects limited microbial activity and reduced nitrogen mineralization under drier environmental conditions. However, the observed increase in the C/P ratio with desertification severity in this region suggests phosphorus accumulation relative to carbon, potentially due to reduced phosphorus uptake by plants or slower phosphorus cycling in degraded soils [33].

The contrasting nutrient dynamics between these regions highlight the critical role of climate in shaping soil nutrient patterns. The pronounced declines in C/N and C/P ratios in the humid monsoon region underscore the vulnerability of these ecosystems to nutrient depletion under intensified desertification, likely driven by accelerated organic matter decomposition and nutrient leaching in moisture-rich environments [31]. Conversely, the stable C/N and elevated C/P ratios in the dry-hot valley indicate greater phosphorus retention, which may reflect slower organic matter turnover and reduced phosphorus mobility under arid conditions [34]. These findings emphasize the need for region-specific soil management strategies that account for climate-driven nutrient dynamics to improve soil fertility and ecosystem stability in rocky desertification-affected landscapes.

### 3.3. Variations in Plant Diversity and Regeneration Across Rocky Desertification Grades and Microclimates in Two Climate Zones

The observed differences in plant diversity and regeneration patterns between the dry-hot valley (Zone 1) and the humid monsoon region (Zone 2) reflect the distinct environmental conditions and ecological processes governing these microclimates. In Zone 1, the dominance of Artemisia in the moderate rocky desertification (MRD) grade aligns with findings that drought-tolerant species tend to dominate in arid environments with reduced soil moisture and nutrient availability [35]. The prevalence of Artemisia in both herbaceous and shrub layers may indicate its strong adaptive capacity in resisting environmental stress and poor soil conditions typical of rocky desertification landscapes [36]. In contrast, Zone 2’s higher species richness and more stable regeneration patterns highlight the role of favorable climatic conditions in supporting vegetation recovery. Humid monsoon climates promote higher soil moisture retention, improving nutrient cycling and enhancing plant growth [37]. The presence of Bidens alongside Artemisia in MRD and non-rocky desertified (NRD) grades further suggests that Zone 2 supports a broader range of species, consistent with studies that report greater ecosystem stability in humid environments [38].

The contrasting responses in these two zones reflect the complex interplay between microclimatic conditions and desertification intensity. While Zone 2 demonstrated greater resilience, the observed decline in plant diversity with increasing desertification severity underscores the vulnerability of both regions to progressive land degradation. The dominance of Vicia under intensive rocky desertification (IRD) conditions in Zone 1 is particularly noteworthy, as legumes like Vicia are known to improve soil nitrogen content and facilitate ecosystem recovery in degraded soils [39]. Such species may play a vital role in stabilizing vegetation under severe environmental stress. These findings emphasize the need for region-specific management strategies that account for microclimatic influences on plant regeneration. In arid environments such as Zone 1, conservation efforts should prioritize drought-tolerant species to maintain vegetation cover and soil stability. Conversely, in humid regions like Zone 2, promoting species diversity through afforestation and sustainable land-use practices may enhance resilience to desertification [40].

### 3.4. Influence of Rocky Desertification and Soil Profile Depth on Soil Active Nitrogen Across Two Climatic Zones

The observed variations in soil active nitrogen dynamics between the dry-hot valley and humid monsoon climate zones support findings from previous studies, which emphasize the climate-dependent nature of soil nutrient cycling in degraded landscapes [41]. In the dry-hot valley, the highest concentrations of ammonium nitrogen (NH_4_^+^-N), nitrate nitrogen (NO_3_^−^-N), microbial biomass nitrogen (MBN), and soil organic nitrogen (SON) were consistently recorded in the intensive rocky desertification (IRD) grade, particularly in the surface soil layer (0–10 cm). This pattern is likely a result of intensified soil erosion, limited vegetation cover, and reduced organic matter input commonly associated with arid conditions, which tend to concentrate nitrogen fractions in surface layers [42]. The dominance of NH_4_^+^-N and NO_3_^−^-N in IRD areas may also be linked to the accumulation of decomposed plant material and reduced nitrogen uptake by vegetation in severely degraded sites [37].

In contrast, nitrogen dynamics in the humid monsoon zone followed a different trend, with the highest concentrations of NH_4_^+^-N, NO_3_^−^-N, MBN, and SON observed in non-rocky desertified (NRD) sites, particularly in the upper soil layer. This suggests that humid conditions promote enhanced organic matter decomposition, microbial activity, and nitrogen mineralization, fostering greater nitrogen availability in less degraded environments [38]. The consistent decline in active nitrogen concentrations with increasing soil depth in both climate zones further emphasizes the importance of surface litter inputs and organic matter accumulation as primary drivers of nitrogen enrichment in shallow soils [43]. Notably, the contrasting nitrogen distribution patterns between the two zones underscore the pivotal role of climate in regulating soil nitrogen cycling processes under rocky desertification. The higher nitrogen content in severely degraded areas of the dry-hot valley aligns with findings from other arid ecosystems, where increased desiccation, reduced leaching, and limited microbial turnover often result in nitrogen being concentrated in surface soils [44]. In contrast, the greater nitrogen availability in NRD sites in the humid monsoon zone is likely due to the region’s higher soil moisture content and greater biological activity, both of which facilitate nitrogen retention in less degraded environments [38].

The results also highlight the significant impact of soil depth on nitrogen distribution, with surface layers consistently exhibiting higher active nitrogen fractions across both climate zones. This aligns with the well-established understanding that microbial activity, root turnover, and organic matter inputs are concentrated in surface horizons, driving enhanced nitrogen cycling in shallow soils [40].

### 3.5. Linkages Between Soil Active Nitrogen Fractions and Soil Physicochemical Characteristics

Soil nitrogen availability is a critical factor influencing ecosystem productivity and nutrient cycling, with physicochemical properties playing a key role in nitrogen transformations [41]. In the dry-hot valley climate zone, the observed positive correlation between soil pH and ammonium nitrogen (NH_4_^+^-N) suggests that alkaline conditions may favor ammonium retention, as higher pH levels reduce ammonia volatilization and promote ammonium stabilization in soil matrices [45]. Conversely, the negative correlation between pH and microbial biomass nitrogen (MBN) indicates that acidic conditions may enhance microbial nitrogen immobilization, which aligns with findings in similar semi-arid ecosystems [44].

The strong positive relationship between soil organic carbon (SOC) and MBN suggests that microbial biomass is largely dependent on organic matter inputs, as SOC serves as a primary energy and nutrient source for microbial communities [46]. Additionally, the significant positive correlations between total nitrogen (TN) and both MBN and soluble organic nitrogen (SON) highlight the integral role of nitrogen pools in microbial activity and soil fertility [47]. These findings support previous studies that emphasize the importance of SOC and TN in regulating microbial nitrogen transformations in arid and semi-arid soils [48]. Similarly, the strong positive correlations between total phosphorus (TP) and both MBN and SON indicate that phosphorus availability may enhance microbial nitrogen assimilation and organic nitrogen turnover [49]. Given that phosphorus is a limiting nutrient in many terrestrial ecosystems, its interaction with nitrogen pools is crucial for sustaining microbial biomass and enzymatic activity [50]. Furthermore, the positive correlation between protease activity and NH_4_^+^-N suggests that proteolytic enzymes play a significant role in ammonification, facilitating nitrogen mineralization and enhancing nitrogen availability in dry-hot valley soils [51]. The observed relationship between NH_4_^+^-N and SON further reinforces the link between ammonification and organic nitrogen transformation processes. The significant positive correlation between MBN and SON suggests that microbial biomass contributes to the turnover of organic nitrogen, converting it into bioavailable forms through decomposition and enzymatic activity [52].

### 3.6. Comparative Analysis of Soil Active Nitrogen Fractions Across Two Climatic Regions

Climate plays a crucial role in shaping soil nitrogen dynamics, particularly under different levels of rocky desertification. Our findings indicate significant variations in soil active nitrogen fractions between the dry-hot valley and humid monsoon climates, highlighting the complex interplay between climate, soil properties, and nitrogen cycling [48]. In non-rocky desertification (NRD) areas, the higher NH_4_^+^-N levels in dry-hot valley climates compared to humid monsoon climates suggest that arid conditions may favor ammonium accumulation. This is likely due to reduced nitrification rates and lower microbial activity under water-limited conditions, which prevent the rapid conversion of NH_4_^+^-N to nitrate (NO_3_^−^-N) [44]. In contrast, the significantly higher NH₃-N levels in humid monsoon climates indicate enhanced volatilization and ammonification processes facilitated by higher soil moisture and temperature [49]. In low (LRD) and moderate (MRD) rocky desertification areas, the higher NH_3_-N concentrations in dry-hot valley climates suggest that nitrogen mineralization rates might be higher in these environments, possibly due to increased microbial enzyme activity under periodic drought conditions [47]. However, the lack of significant differences in NH_3_-N levels in intermediate rocky desertification (IRD) areas suggests that the effects of climate on nitrogen transformations might be buffered by other factors, such as soil texture and organic matter content [36].

Microbial biomass nitrogen (MBN) levels followed a distinct pattern across the two climatic regions. In NRD areas, the significantly higher MBN in humid monsoon climates implies that microbial communities thrive under more favorable moisture conditions, where nitrogen mineralization and assimilation processes are enhanced [51]. Conversely, in LRD, MRD, and IRD areas, the higher MBN in dry-hot valley climates suggests that microbial adaptation strategies, such as enhanced nitrogen retention and drought-resistant microbial populations, play a role in sustaining microbial biomass in arid conditions [25]. Soluble organic nitrogen (SON) concentrations exhibited a consistent pattern across the desertification gradient. The significantly higher SON levels in dry-hot valley climates in NRD and LRD areas suggest that arid environments promote the accumulation of organic nitrogen compounds due to slower decomposition rates and limited microbial uptake [50]. However, the absence of significant differences in SON levels in MRD and IRD areas between the two climates indicates that factors such as plant litter input, microbial diversity, and soil structure may regulate SON dynamics beyond climatic influences alone [53].

Overall, these findings highlight the differential responses of soil nitrogen fractions to climate conditions and desertification gradients. The distinct patterns observed in NH_4_^+^-N, NH_3_-N, MBN, and SON emphasize the need to consider both climatic and ecological factors when assessing nitrogen cycling in degraded landscapes. Future research should explore microbial community composition and enzyme activity to further elucidate the mechanisms driving these nitrogen transformations in varying climatic conditions.

## 4. Materials and Methods

### 4.1. Study Sites

This study was conducted in two ecologically distinct regions of Guizhou Province, Southwest China: the Beipan River region at the Guanling–Zhenfeng junction (25°39′12″ to 25°41′09″ N, 105°38′59″ to 105°40′27″ E) and Puding County in Anshun City (26°17′12″ to 26°22′09″ N, 105°44′53″ to 105°45′19″ E). Despite differences in climate and topography, these sites provide valuable insights into the impacts of environmental factors and human activities on Guizhou’s karst ecosystems.

The Guanling–Zhenfeng junction, located along the Beipan River, has an average elevation of approximately 1270 m. This area experiences a dry-hot valley climate typical of the southern subtropics, characterized by distinct wet and dry seasons. The average annual temperature is 16.2 °C, with an average annual rainfall of about 1458 mm. Geologically, the region is primarily composed of limestone. The local community, adapting to challenging environmental conditions such as drought, water scarcity, and shallow soils, cultivates drought-tolerant cash crops like dragon fruit, sugarcane, prickly ash, and loquat. Since the 1980s, management efforts have been implemented in this important karst area, recognized both nationally and globally. However, the region continues to face severe rocky desertification, driven by its fragile geology and unsustainable human activities, which pose significant challenges to local economic development.

Puding County, located at an average altitude of 1205 m, has an average annual temperature of 15.1 °C and receives approximately 1396.9 mm of rainfall each year. The geology here is also dominated by limestone, with soil primarily consisting of brown limestone weathered from carbonate rocks. This area experiences a typical humid monsoon climate of the northern subtropics and is one of the three major rainfall centers in Guizhou Province. However, the region’s unique topography and geomorphology have been negatively impacted by a combination of climate factors and human activities, leading to the degradation of its once-productive agricultural ecosystem. Currently, the region faces severe ecological challenges, including intensified rocky desertification, increased bedrock exposure, and widespread vegetation degradation.

### 4.2. Experimental Design and Soil Sampling Collection

In March 2021, a nested experimental design was employed to investigate soil properties across two distinct climate regions within the karst ecosystem: the dry-hot valley and the humid monsoon. These climate regions were specifically chosen to examine their effects on soil characteristics under varying degrees of rocky desertification. Four grades of rocky desertification—non-rocky desertification (NRD), light rocky desertification (LRD), moderate rocky desertification (MRD), and intense rocky desertification (IRD)—were classified according to established criteria and nested within the two climate regions. The bedrock exposure rates for these grades were as follows: NRD (30% exposure), LRD (30–50% exposure), MRD (50–70% exposure), and IRD (greater than 70% exposure) (see Table 4).

For each degradation grade, three quadrats of 50 × 50 m were established, ensuring a minimum of 200 m between quadrats to maintain spatial independence. Soil samples were collected from three depths—0–10 cm (A), 10–20 cm (B), and 20–30 cm (C)—within each quadrat. Each sample consisted of five subsamples, collected in an ‘S’ pattern to ensure a comprehensive representation of the area. This resulted in a total of 180 soil samples, calculated as 2 climate regions × 4 degradation grades × 3 depths × 3 replicates × 5 subsamples.

Plant diversity in the dry-hot valley (Zone 1) and the humid monsoon (Zone 2) were assessed using field surveys across four rocky desertification grades: non-rocky desertification (NRD), light rocky desertification (LRD), moderate rocky desertification (MRD), and intense rocky desertification (IRD). Quadrats of 1 m × 1 m for herbaceous plants and 5 m × 5 m for shrubs were established at each site, with three replicates per grade. Within each quadrat, species were identified to the species level, and the abundance of herbaceous plants and percentage cover of shrubs were recorded. Species richness was calculated as the total number of species, while regeneration was assessed by counting seedlings and juvenile plants. Diversity indices, including the Shannon–Wiener index (H′) and Simpson’s index (D), were used to evaluate species diversity and evenness. Data were analyzed statistically to compare diversity metrics across zones and desertification grades. This method allowed for a comprehensive evaluation of plant diversity and regeneration, highlighting differences between the two microclimates and their responses to rocky desertification.

In the laboratory, samples intended for soil enzyme activity analysis were sieved through a 2 mm mesh and stored at 4 °C to preserve biological integrity. The remaining samples were air-dried and sieved to 0.149 mm for subsequent physicochemical analyses. This systematic sampling approach ensures that the soil samples accurately reflect the diverse environmental conditions present across the different grades of rocky desertification. By employing a split-plot design, this study effectively assesses the interactions between degradation grades and soil depth, providing valuable insights into soil dynamics and environmental variability in these ecosystems.

### 4.3. Soil Sampling Analysis

In this study, key soil properties and chemical contents were analyzed using established laboratory techniques. Soil moisture content (SMC), bulk density (BD), and soil porosity (SP) were measured using the ring knife method. Soil organic carbon (SOC) was assessed via the potassium dichromate oxidation method, while total nitrogen (TN) was determined using the semi-micro Kjeldahl method. The total phosphorus (TP) content was measured using the molybdenum blue colorimetric method [54]. Soil pH was evaluated by mixing soil and water in a 1:2.5 ratio, allowing the mixture to stand for 30 min before measuring the pH with a glass electrode. For enzyme activity, soil urease and protease levels were quantified using indophenol blue colorimetric ultraviolet spectrophotometry and the ninhydrin colorimetric method [55], respectively. Ammonium nitrogen (NH_4_^+^-N) concentration was determined using indophenol blue colorimetric ultraviolet spectrophotometry [16]. In this procedure, NH_4_^+^-N ions were extracted from the soil using a 2 mol/L KCl solution, then reacted with hypochlorite and phenol in a strong alkaline medium to form a stable indophenol blue solution.

Nitrate nitrogen (NO_3_^−^-N) content was assessed by the phenol disulfonic acid colorimetric method, in which NO_3_⁻-N reacts with phenol disulfonic acid to form a yellow-colored solution under anhydrous conditions [16]. Microbial biomass nitrogen (MBN) was quantified using the fumigation–extraction ninhydrin colorimetric method. In this process, microbial residues were destroyed by fumigation and extracted using K_2_SO_4_, followed by heating with ninhydrin reagent to produce a purple color. The difference in nitrogen content between fumigated and non-fumigated samples indicates the microbial biomass nitrogen content.

### 4.4. Data Analysis

To assess the soil physicochemical properties and active nitrogen (AN) fractions across different grades of rocky desertification in both the dry-hot valley and humid monsoon climate regions, a three-way Analysis of Variance (ANOVA) was conducted to evaluate the effects of rocky desertification levels, climate zones, and soil depth, as well as their interactions. This approach allowed for the identification of significant differences among the means of various treatment groups. Additionally, the Wilcoxon signed-rank test was used to compare soil active nitrogen fractions across different grades of rocky desertification and three soil layers within the two climatic regions, providing a non-parametric method to account for potential non-normality in the data distribution. All graphical representations and visual analyses were generated using Origin 2021 Pro, ensuring clear and precise depiction of the results. Preliminary data processing was carried out in Excel 2010, while comprehensive statistical analysis was performed using SPSS 26.0 (SPSS Inc., Chicago, IL, USA).

## 5. Conclusions

This study identified significant differences in soil properties, nitrogen dynamics, and plant regeneration across different rocky desertification grades and climate zones (dry-hot valley and humid monsoon). Although soil organic carbon (SOC), total nitrogen (TN), and total phosphorus (TP) were higher in severely desertified areas, these increases likely reflect reduced plant uptake and organic matter decomposition rather than improved soil fertility. Meanwhile, while soil moisture content (SMC) and pH varied with climate and desertification severity. In the dry-hot valley, ammonium nitrogen (NH_4_^+^-N) was consistently elevated, whereas nitrate nitrogen (NO_3_^−^-N) declined with increasing desertification. Microbial biomass nitrogen (MBN) and soil organic nitrogen (SON) showed complex trends, with higher values in less desertified areas. In contrast, the humid monsoon climate exhibited lower NH₄⁺-N in desertified regions, with distinct NO_3_^−^-N and MBN patterns.

Plant diversity and regeneration were significantly influenced by desertification grades and climate conditions. In the dry-hot valley (Zone 1), Artemisia dominated herbaceous regeneration, especially in Moderate Rock Desertification (MRD) areas. Shrub regeneration also showed a strong Artemisia presence, particularly in Light Rock Desertification (LRD) zones. Conversely, the humid monsoon climate (Zone 2) supported greater plant diversity, with Artemisia and Bidens abundant in MRD and Non-Rock Desertification (NRD) areas. As desertification intensified, both plant diversity and regeneration declined in two zones; however, Zone 2 exhibited relatively higher species richness and resilience, suggesting that local environmental conditions may buffer some of the negative impacts of severe rocky desertification. These findings underscore the influence of climate and desertification severity on soil and vegetation dynamics. Targeted management strategies are needed to mitigate soil degradation, enhance nutrient availability, and promote biodiversity. This study highlights the potential for ecosystem restoration in monsoon climates, where favorable conditions may support more effective rehabilitation of desertified landscapes. Sustainable land-use practices should prioritize leveraging these ecological advantages to improve long-term ecosystem resilience.

## Figures and Tables

**Table 1:** Soil Physicochemical Properties and Enzyme Activity across Different Grades of Rocky Desertification and Soil Depths.

Dry-Hot Valley Climate Zone								
Rocky Desertification Grades	Depth	SMC (%)	pH	SOC (g/kg)	TN (g/kg)	TP (g/kg)	Urease (mg/g)	Protease (mg/g)
	A	18.64 ± 0.22 Ca	8.24 ± 027 Aa	13.65 ± 0.45 Aa	1.84 ± 0.09 Da	0.35 ± 0.08 Ca	1.06 ± 0.10 Bc	0.04 ± 0.00 Ab
NRD	B	19.89 ± 1.0.9 Ba	8.22 ± 0.02 Aa	7.18 ± 0.67 Ab	1.38 ± 0.08 Cb	0.36 ± 0.05 Ca	0.38 ± 0.07 Ab	0.04 ± 0.00 Aab
	C	21.27 ± 1.78 Ba	8.12 ± 0.12 Aa	4.40 ± 0.54 Ac	1.14 ± 0.04 Cc	0.29 ± 0.03 Ca	0.12 ± 0.05 Aa	0.04 ± 0.00 Ba
	A	20.32 ± 0.34 BCa	8.13 ± 0.06 ABa	26.79 ± 1.30 Ba	2.49 ± 0.11 Ca	0.92 ± 0.15 Ba	1.93 ± 0.27 Ca	0.05 ± 0.01 Ab
LRD	B	20.83 ± 0.99 Ba	8.08 ± 0.02 Ba	21.97 ± 1.68 Bb	2.05 ± 0.05 Bb	0.84 ± 0.11 Ba	2.09 ± 0.68 Ba	0.04 ± 0.01 Ab
	C	21.60 ± 1.03 Ba	8.01 ± 0.07 Aa	19.10 ± 0.07 Bc	1.97 ± 0.04 Bb	0.72 ± 0.04 Ba	1.38 ± 0.37 Ca	0.02 ± 0.00 Aa
	A	23.48 ± 0.89 ABb	7.84 ± 0.07 BCa	19.20 ± 1.95 Ca	2.15 ± 0.036 Ba	0.81 ± 0.26 BCa	0.50 ± 0.09 Ab	0.06 ± 0.01 Ab
MRD	B	24.17 ± 0.06 Aab	7.46 ± 0.06 Cb	16.79 ± 2.01 Cab	1.95 ± 0.08 Bb	0.79 ± 0.24 Ba	0.26 ± 0.07 Aa	0.05 ± 0.01 Aab
	C	24.24 ± 0.49 ABa	7.37 ± 0.06 Bb	13.71 ± 1.57 Cb	1.83 ± 0.09 Bb	0.67 ± 0.12 Ba	0.14 ± 0.01 Aa	0.04 ± 0.01 Ba
	A	25.90 ± 2.47 Aa	7.52 ± 0.22 Ca	30.83 ± 1.67 Da	3.32 ± 0.05 Aa	1.92 ± 0.24 Aa	0.77 ± 0.08 ABa	0.05 ± 0.01 Aab
IRD	B	26.18 ± 1.40 Aa	7.43 ± 0.04 Cab	30.72 ± 1.31 Da	3.04 ± 0.05 Ab	1.79 ± 0.11 Aa	0.75 ± 0.07 Aa	0.05 ± 0.03 Ab
	C	27.08 ± 1.70 Aa	7.36 ± 0.04 Bb	25.30 ± 2.61 Db	2.74 ± 0.11 Ac	1.61 ± 0.24 Aa	0.71 ± 0.07 Ba	0.02 ± 0.00 Aa
Humid Monsoon Climate Zone								
	A	16.18 ± 3.15 Aa	7.58 ± 0.02 Ca	42.06 ± 4.25 Ba	3.47 ± 0.22 Ba	1.05 ± 0.27 Ba	1.18 ± 0.29 Ba	0.06 ± 0.01 Cb
NRD	B	18.7 ± 4.66 Aa	7.61 ± 0.10 Ca	37.94 ± 5.44 Ba	3.37 ± 0.22 Ba	0.92 ± 0.19 Ba	1.04 ± 0.07 Ca	0.04 ± 0.01 Cab
	C	18.85 ± 2.72 Aa	7.65 ± 0.05 Ca	37.89 ± 8.25 Ba	2.97 ± 0.64 Ba	0.83 ± 0.17 Ba	0.86 ± 0.16 Ca	0.04 ± 0.01 Ba
	A	17.24 ± 0.06 Aa	6.04 ± 0.35 Aa	20.36 ± 3.31 Aa	1.68 ± 0.20 Ab	0.37 ± 0.04 Ab	0.73 ± 0.01 Ac	0.05 ± 0.01 Bcb
LRD	B	18.08 ± 0.58 Aa	6.2 ± 0.41 Aa	17.03 ± 0.09 Aa	1.32 ± 0.09 Aab	0.31 ± 0.01 Aab	0.22 ± 0.08 Ab	0.03 ± 0.00 Bca
	C	19.41 ± 2.00 Aa	6.38 ± 0.44 Aa	16.96 ± 0.09 Aa	1.09 ± 0.25 Aa	0.27 ± 0.05 Aa	0.11 ± 0.02 Aa	0.03 ± 0.00 Ba
	A	19.18 ± 1.23 Aa	5.99 ± 0.05 Aa	21.98 ± 4.34 Aa	1.88 ± 0.29 Aa	0.59 ± 0.09 Aa	0.51 ± 0.07 Ac	0.04 ± 0.02 ABb
MRD	B	19.88 ± 0.23 Aa	6 ± 0.15 Aa	20.91 ± 3.86 Aa	1.56 ± 0.58 Aa	0.42 ± 0.17 Aa	0.36 ± 0.04 Bb	0.03 ± 0.01 ABab
	C	21.39 ± 1.98 ABa	6.17 ± 0.20 Aa	20.47 ± 5.54 Aa	1.37 ± 0.61 Aa	0.38 ± 0.19 Aa	0.13 ± 0.02 Aa	0.01 ± 0.00 Aa
	A	25.85 ± 1.47 Ba	6.94 ± 0.18 Ba	35.67 ± 1.92 Ba	3.74 ± 0.32 Ba	0.94 ± 0.05 Ba	0.68 ± 0.11 Ab	0.02 ± 0.00 Ab
IRD	B	26.06 ± 4.12 Ba	6.95 ± 0.09 Ba	34.51 ± 3.92 Ba	3.44 ± 0.60 Ba	0.82 ± 0.16 Ba	0.43 ± 0.09 Ba	0.02 ± 0.00 Aab
	C	27.26 ± 3.13 Ba	7.02 ± 0.04 Ba	31.21 ± 1.94 Ba	3.18 ± 0.38 Ba	0.76 ± 0.12 Ba	0.35 ± 0.10 Ba	0.01 ± 0.00 Aa

Note: The numerical values represent the mean ± standard deviation for each sample analysis repetition. One-way ANOVA and LSD multiple comparisons were performed. Capital letters indicate significant differences among the rocky desertification grades within the same soil layer, while lowercase letters indicate significant differences between soil layers within the same rocky desertification grade (*p* < 0.05). A, B, and C correspond to soil depths of 0–10 cm, 10–20 cm, and 20–30 cm, respectively. Capital letters (A, B, C) indicate significant differences among the four grades of rocky desertification, while lowercase letters (a, b, c) indicate significant differences within the soil layers A, B, and C.

**Table 2:** Soil C/N, C/P, and N/P Ratios Across Four Grades of Rocky Desertification and Three Soil Layers in Two Regions.

Dry-Hot Valley Climate Zone				
Rocky Desertification Grades	Soil Depth	C/N	C/P	N/P
	A	7.42 ± 0.38 Ac	40.69 ± 9.10 Aa	5.45 ± 1.00 Aa
NRD	B	5.22 ± 0.20 Ab	20.37 ± 2.60 ABb	3.90 ± 0.43 Cb
	C	3.85 ± 0.38 Aa	15.40 ± 3.85 Ab	3.96 ± 0.58 Cb
	A	10.77 ± 0.41 Cb	29.59 ± 4.04 ABa	2.76 ± 0.47 Ba
LRD	B	10.71 ± 0.75 Cb	26.54 ± 5.22 Ba	2.47 ± 0.32 ABa
	C	9.68 ± 0.20 Ca	26.52 ± 1.56 Ca	2.74 ± 0.21 Ba
	A	8.91 ± 0.81 Ba	25.64 ± 9.31 Ba	2.84 ± 0.82 Ba
MRD	B	8.6 ± 0.68 Ba	22.21 ± 5.34 ABa	2.59 ± 0.63 Ba
	C	7.51 ± 0.81 Ba	20.80 ± 1.81 Ba	2.80 ± 0.46 Ba
	A	9.3 ± 0.62 Ba	16.26 ± 2.86 Ba	1.74 ± 0.21 Ba
IRD	B	10.11 ± 0.54 Ca	17.17 ± 0.33 Aa	1.70 ± 0.12 Aa
	C	9.22 ± 0.73 Ca	15.78 ± 1.42 Aa	1.72 ± 0.20 Aa
Humid Monsoon Climate Zone				
	A			
NRD	B	11.2 ± 0.93 Aa	42.82 ± 13.78 Aa	3.78 ± 0.97 Aa
	C	12.81 ± 1.18 Aa	48.78 ± 21.81 Aa	3.78 ± 1.50 Aa
	A	12.12 ± 0.85 Ba	54.47 ± 4.31 Aa	4.50 ± 0.45 Ba
LRD	B	12.93 ± 0.86 Aa	54.74 ± 2.36 Aa	4.24 ± 0.32 Aa
	C	16.12 ± 3.71 Aa	63.08 ± 12.04 Aa	3.95 ± 0.40 Aa
	A	11.74 ± 1.93 Ba	37.78 ± 8.49 Aa	3.19 ± 0.24 Aa
MRD	B	14.67 ± 5.46 Aa	54.83 ± 20.67 Aa	3.75 ± 0.37 Aa
	C	16.25 ± 5.49 Aa	62.27 ± 28.60 Aa	3.74 ± 0.42 Aa
	A	9.56 ± 0.49 Aa	37.88 ± 0.30 Aa	3.97 ± 0.24 ABa
IRD	B	10.13 ± 0.73 Aa	42.60 ± 3.75 Aa	4.21 ± 0.28 Aa
	C	9.87 ± 0.74 Aa	41.79 ± 5.97 Aa	4.22 ± 0.29 Aa

Note: Soil C/N, C/P, and N/P ratios are presented for both climate regions (dry-hot valley and humid monsoon) across the three soil layers: NRD (non-rocky desertification), LRD (light rocky desertification), MRD (moderate rocky desertification), and IRD (intense rocky desertification). Capital letters (A, B, C) indicate significant differences among the four grades of rocky desertification. Lowercase letters (a, b, c) indicate significant differences within the soil layers A, B, and C.

**Table 3:** Regeneration Statistics of Plant Species Across Four Grades of Rocky Desertification in Two Microclimates.

Genus (Species)	NRD (Herb) Zone 1	NRD (Shrub) Zone 1	LRD (Herb) Zone 1	LRD (Shrub) Zone 1	MRD (Herb) Zone 1	MRD (Shrub) Zone 1	IRD (Herb) Zone 1	IRD (Shrub) Zone 1	NRD (Herb) Zone 2	NRD (Shrub) Zone 2	LRD (Herb) Zone 2	LRD (Shrub) Zone 2	MRD (Herb) Zone 2	MRD (Shrub) Zone 2	IRD (Herb) Zone 2	IRD (Shrub) Zone 2
Artemisia (*Artemisia*)	24 (20%)	5 (33%)	48 (24%)	5 (14%)	69 (52%)	1 (4%)	5 (7%)	4 (27%)	5 (7%)	0	0	0	5 (10%)	0	0	0
Rubia (*Rubia*)	5 (4%)	9 (25%)	0	0	0	0	1 (7%)	4 (27%)	0	0	0	0	0	0	0	0
Vicia (*Vicia*)	14 (11%)	1 (3%)	0	0	25 (19%)	1 (4%)	23 (33%)	0	0	0	0	0	14 (20%)	1 (6%)	0	0
Adiantum (*Adiantum*)	8 (7%)	1 (3%)	0	0	0	1 (4%)	2 (3%)	1 (7%)	0	0	0	0	8 (13%)	0	0	0
Amaranthus (*Amaranthus*)	3 (2%)	4 (11%)	0	0	0	0	9 (13%)	4 (27%)	0	0	0	0	3 (5%)	4 (15%)	0	0
Bidens (*Bidens*)	10 (8%)	1 (3%)	0	0	13 (10%)	0	1 (1%)	2 (13%)	2 (3%)	1 (3%)	0	0	8 (10%)	0	0	0
Ivy (*Ivy*)	3 (2%)	2 (6%)	5 (2%)	0	6 (5%)	2 (13%)	7 (10%)	0	0	2 (3%)	5 (6%)	0	3 (8%)	0	2 (3%)	
Stachys (*Stachys*)	2 (2%)	5 (14%)	0	0	11 (8%)	3 (12%)	9 (13%)	4 (27%)	0	5 (10%)	0	0	12 (10%)	0	0	0
Taraxacum (*Taraxacum*)	10 (8%)	1 (3%)	10 (8%)	0	4 (3%)	1 (4%)	5 (7%)	0	0	0	5 (5%)	0	10 (15%)	0	5 (5%)	0
Gynura (*Gynura*)	10 (8%)	0	0	5 (2%)	0	5 (19%)	5 (7%)	2 (13%)	0	0	0	0	10 (8%)	0	0	0
Epimedium (*Epimedium*)	2 (2%)	0	0	1 (4%)	0	2 (8%)	0	4 (27%)	0	0	0	0	2 (5%)	0	0	0
Phyllitis (*Phyllitis*)	16 (13%)	0	23 (0%)	9 (5%)	0	4 (15%)	5 (7%)	2 (13%)	0	0	0	0	16 (20%)	0	0	0
Clematis (*Clematis*)	3 (2%)	0	0	0	0	1 (4%)	0	4 (27%)	0	0	0	0	3 (5%)	0	0	0
Total Herbs	101		60		30		31		15		5		45		3	
Total Shrubs		73		56		14		14								

Note: NRD (Non-rocky desertification): Areas without any significant rocky desertification. LRD (light rocky desertification): Areas with minimal rocky exposure and some vegetation. MRD (moderate rocky desertification): Areas with moderate rocky outcrops and reduced vegetation cover. IRD (intense rocky desertification): Severely rocky areas with limited soil and vegetation. Zone 1: dry-hot valley; Zone 2: humid monsoon.

**Table 4:** The Classification Standards for Different Rocky Desertification Grades.

Rocky Desertification Grades	Exposed Rate of Bedrock	Soil Depth Condition	Vegetation Characteristics
NRD	0~30%	The soil layer is deep and easy to collect	Trees are dominant, shrubs and herbs are secondary
LRD	30~50%	The soil layer is shallow and it is difficult to collect samples	Few trees, mainly shrubs and herbs
MRD	50~70%	The soil layer is shallow and it is difficult to collect samples	There are almost no trees, mainly shrubs and herbs
IRD	>70%	The soil layer is shallow and it is very difficult to collect samples	Mainly herbs and shrubs

## Data Availability

The data presented in this study are available upon request from the corresponding authors.
